# The International Hahnemannian Association

**Published:** 1899-08

**Authors:** Wilson A. Smith


### THE INTERNATIONAL HAHNEMANNIAN ASSOCIATION.

This Society held a meeting at Niagara Falls the last of June and we stopped on our return from the American Institute for a short visit among those who claim to stand for pure Homœopathy. It was a matter of much disappointment to find at the meeting less than a baker's dozen, and the question very naturally arose in one's mind if the Society had not outlived its usefulness.

Twelve members are not much of a showing for an international affair. It strikes the outsider that if they desire to convert one, it will require more enthusiasm on the part of those who so loudly proclaim their purity and a greater effort to be present at the meeting. The fact really is that what good, if any, could have been done by the splitting away from the parent institution, has been accomplished and the proper thing for them to do is to return to the folds of the old Association and use what little influence they possess in educating the vast membership into a higher ideal of prescribing.

Every one there was a firm believer in the Homœopathy as taught and practiced by them, and it would seem, if they wished to convert others, that they should not wrap their cloaks about themselves and keep away from those in darkness, but that they should go among the heathens and carry the gospel of purity where it will have some opportunity of reaching those who know but little of the effects of using remedies as they believe they should be used. We believe there is a good text for them in the passage of Scripture where it says one should not hide his light under a bushel. We hope to see the whole Association members of the old Institute again, for it is there that they will find the heathens.

This is written in the spirit of good will. Not a desire to find fault enters into the thought which prompts the writing, but a desire, if they are right, that the truths they stand so strongly for shall be spread before all the people. We believe that a majority of those who belong to the I. H. A. feel about this as does *The Medical Visitor.*—*Wilson A. Smith, M. D., in Medical Visitor.*

